# Improvement of treatment plan quality with modified fixed field volumetric modulated arc therapy in cervical cancer

**DOI:** 10.1002/acm2.14479

**Published:** 2024-07-20

**Authors:** Sirawat Jindakan, Ekkasit Tharavichitkul, Anirut Watcharawipha, Wannapha Nobnop

**Affiliations:** ^1^ Medical Physics Program Department of Radiology Faculty of Medicine Chiang Mai University Chiang Mai Thailand; ^2^ Department of Radiology Faculty of Medicine The Division of Radiation Oncology Chiang Mai University Chiang Mai Thailand

**Keywords:** auto field, cervical cancer, modified fixed field, volumetric modulated arc therapy

## Abstract

**Purpose:**

This study aims to introduce modified fixed field volumetric modulated arc therapy (MF‐VMAT) which manually opened the field size by fixing the jaws and comparing it to the typical planning technique, auto field volumetric modulated arc therapy (AF‐VMAT) in cervical cancer treatment planning.

**Methods and materials:**

Previously treated twenty‐eight cervical cancer plans were retrospectively randomly selected and replanned in this study using two different planning techniques: AF‐VMAT and MF‐VMAT, resulting in a total of fifty‐six treatment plans. In this study, we compared both planning techniques in three parts: (1) Organ at Risk (OARs) and whole‐body dose, (2) Treatment plan efficiency, and (3) Treatment plan accuracy.

**Results:**

For OARs dose, bowel bag (*p*‐value = 0.001), rectum (*p*‐value = 0.002), and left femoral head (*p*‐value = 0.001) and whole‐body (*p*‐value = 0.000) received a statistically significant dose reduction when using the MF‐VMAT plan. Regarding plan efficiency, MF‐VMAT exhibited a statistically significant increase in both number of monitor units (MUs) and control points (*p*‐values = 0.000), while beam‐on time, maximum leaf travel, average maximum leaf travel, and maximum leaf travel per gantry rotation were statistically significant decreased (*p*‐values = 0.000). In terms of plan accuracy, the average gamma passing rate was higher in the MF‐VMAT plan for both absolute dose (AD) (*p*‐value = 0.001, 0.004) and relative dose (RD) (*p*‐value = 0.000, 0.000) for 3%/3 and 3%/2 mm gamma criteria, respectively.

**Conclusion:**

The MF‐VMAT planning technique significantly reduces OAR doses and decreases the spread of low doses to normal tissues in cervical cancer patients. Additionally, this planning approach demonstrates efficient plans with lower beam‐on time and reduced maximum leaf travel. Furthermore, it indicates higher plan accuracy through an increase in the average gamma passing rate compared to the AF‐VMAT plan. Consequently, MF‐VMAT offers an effective treatment planning technique for cervical cancer patients.

## 1 INTRODUCTION

Cervical cancer ranks as the fourth most prevalent cancer and contributor to cancer‐related fatalities among women globally, following breast, lung, and colorectal cancers.^1^ On a worldwide scale, cervical cancer typically occurs at an average age of 53, with ages ranging from 44 to 68.^1^ Consequently, it remains a significant public health concern primarily impacting women in their middle‐age, especially in countries with fewer resources.^1^

Radical hysterectomy with pelvic lymph node assessment is the primary treatment approach for early‐stage cervical cancer (classified as FIGO stage IB1–IIA), whereas definitive radiotherapy plays an important role in patients who are unsuitable candidates for surgery, both resulting in comparable survival rates.^2^, ^3^, ^4^

Adjuvant radiotherapy is an important modality in the treatment of endometrial and cervical cancers.^5^ It is recommended based on histopathological risk factors. In addition to treatment methods in surgery and radiotherapy progress, adjuvant radiotherapy should be personalized within the intermediate‐risk clinical setting.^6^ Moreover, the addition of concurrent cisplatin‐based chemotherapy to radiation therapy shows enhancement in progression‐free and overall survival (OS) for high‐risk early‐stage cervical cancer.^6^

Intensity modulated radiation therapy (IMRT) is a radiotherapy technique that allows for the delivery of highly conformal dose distribution while minimizing radiation dose to organs at risk (OARs).^7^ Volumetric modulated arc therapy (VMAT) is a novel treatment technique that provides equivalent target coverage compared to conventional IMRT while significantly reducing the dose to OARs including bowel, rectum, bladder, and femoral head.^8^ Furthermore, VMAT enhances conformity and homogeneity indices to the planning target volume (PTV), along with reducing average monitor units (MUs) and shortening delivery time.^8^ However, a general drawback of VMAT is the concern regarding low‐dose exposure to normal tissues.^9^ Due to the 5‐year OS rate for both early‐stage and locally advanced‐stage cervical cancer patients being more than 60%,^10^, ^11^, ^12^, ^13^ the radiation‐induced toxicity for many OARs in gynecological cancer following pelvic radiotherapy is a significant concern. In addition to the primary morbidities, which are typically genitourinary (GU) and gastrointestinal (GI) complications rated grade ≥3, following pelvic irradiation.^14^, ^15^ Furthermore, concurrent chemoradiotherapy (CCRT) with external beam radiotherapy (EBRT), followed by intracavitary brachytherapy (ICBT) is the standard treatment for locally advanced cervical cancer (LACC) patients.^7^ Definitive radiotherapy patients are typically treated with chemoradiotherapy followed by ICBT,^16^, ^17^ while adjuvant radiotherapy patients with vaginal and/or parametrial positive disease can also receive a boost with ICBT.^17^ Therefore, reducing the dose of OARs during EBRT for these patients is important to avoid the risk of complications after the boost with ICBT.

While several studies have reported the advantages of half‐beam VMAT, which eliminates the mechanical limitations of Varian multileaf collimators (MLCs) in treating large PTVs of pelvic cancer,^18^, ^19^, ^20^, ^21^ research on half‐beam VMAT using Elekta MLCs has been limited. Although Elekta LINACs don't have mechanical limitations of MLCs, other aspects like the reduced MLCs speed by half‐beam VMAT are also interesting and have the potential to yield better dosimetric results and plan delivery accuracy relative to previous studies.^22^, ^23^, ^24^, ^25^, ^26^, ^27^ The half‐beam VMAT technique is a specialized planning technique in radiation therapy used to deliver precise radiation doses to the PTV while minimizing exposure to OARs. Half‐beam VMAT consists of two coplanar arcs, one rotating clockwise and the other counterclockwise. Both arcs contain one half‐beam field, with either the left or right side shielded by the jaws.^18^, ^19^, ^20^, ^21^ Although, the Monaco treatment planning system has the multiple arc‐per‐beam (APB) option, this parameter allows the optimization algorithm to automatically split fluence while using a single collimator angle and allowing the gantry to rotate clockwise and counterclockwise without stopping radiation delivery.^28^ Nevertheless, one study reported that in the PTV group of cervical cancer patients, the 2 arc‐per‐beam (2APB) setting significantly reduced MUs and control points and slightly improved CI. However, there was no significant difference in OARs dose sparing, including the rectum, bladder, and small bowel.^29^ These limitations motivate the development of an improved treatment planning technique to address the drawbacks associated with VMAT, reduce the dose to OARs, decrease the rate of radiation‐induced toxicities, and improve the quality of life for the patients. The objective of this study is to introduce modified fixed field volumetric modulated arc therapy (MF‐VMAT), which manually opened the field size by fixing the jaws and comparing it to the typical planning technique, auto field volumetric modulated arc therapy (AF‐VMAT) in three parts: (1) OARs and whole‐body dose, (2) Treatment plan efficiency, and (3) Treatment plan accuracy for cervical cancer treatment planning.

## 2 METHODS AND MATERIALS

### 2.1 Patient selection, CT simulation, and target delineation

Twenty‐eight cervical cancer patients treated at our center from July 2023 to January 2024 were retrospectively randomly selected. These patients were divided into two groups including adjuvant and definitive radiotherapy. The characteristics of these patients are presented in Table 1. All patients had cancer ranging from stage IB1 to IVA. The patients underwent CT simulation with a bladder preparation. Non‐contrast pelvic CT simulations were performed with a slice thickness of 5 mm, with patients in a supine position and ankles locked in immobilization. The clinical target volume (CTV) included the gross tumor volume (GTV) and the regional lymph nodes affected by cancer spread. The PTV was created by expanding 0.5 cm from the CTV in all directions. This study was granted an ethics exemption by the Research Ethics Committee of the Faculty of Medicine, Chiang Mai University (study code: RAD‐2566‐0554/research ID: 0554).

**TABLE 1:** Characteristics of patients and tumors.

Characteristics	Description
Age	30 to 89
Stage	IB1 = 2, IB2 = 2, IB3 = 5, IIB = 10, IIIB = 3, IIIC1 = 4, IIIC2 = 1, IVA = 1
Pathology	AC = 9, SCC = 18, ASCC = 1
Treatment method	Postoperative RT = 14 RT alone = 4 Combine Rx = 10
Treatment aim	Adjuvant = 14 Definitive = 14

Abbreviations: AC, adenocarcinoma; ASCC, adenosquamous carcinoma; RT, radiation therapy; Rx, treatment regimens; SCC, squamous cell carcinoma.

### 2.2 Planning techniques

All twenty‐eight plans were replanned using two different planning techniques, Auto field VMAT (AF‐VMAT) plans and modified fixed field VMAT (MF‐VMAT) plans. Both treatment planning techniques involved two full coplanar arcs, with one rotating clockwise and the other counterclockwise, starting the gantry angle at 180°, and each arc covering 360° with the couch angle at 0°. The increment gantry value (sector spacing) was set at 30° in full‐ arc of VMAT with the equal 12 sectors. The number of sectors is determined by dividing the total arc degree by the increment gantry value. In each sector, MLC leaves only move unidirectionally and may generate many control points. Each plan utilized a maximum of one arc, a maximum of 250 control points per arc, a minimum segment width of 0.5 cm, and high fluence smoothing.

Several studies of dual‐arc VMAT have shown better benefits compared to single‐arc VMAT and the IMRT technique. Two coplanar arcs in VMAT demonstrate the best performance in terms of CI, HI, and OARs sparing, with fewer MUs and decreased treatment time compared to 9‐field IMRT in cervical cancer patients.^30^ Additionally, dual‐arc VMAT plans with 6 MV photon energy are a good choice for treating cervical cancer, delivering highly homogeneous and conformal plans with superior target coverage and better OARs sparing.^31^ In VMAT delivery, the collimator angle is typically rotated to reduce interleaf leakage and the tongue‐and‐groove effect.^32^, ^33^ Previous study has reported that using two full coplanar arcs with a collimator angle of 35° provides the most consistent dosimetric results, better CI, and more sparing of OARs in gynecological cancer compared to IMRT and single‐arc VMAT.^34^

**FIGURE 1:**
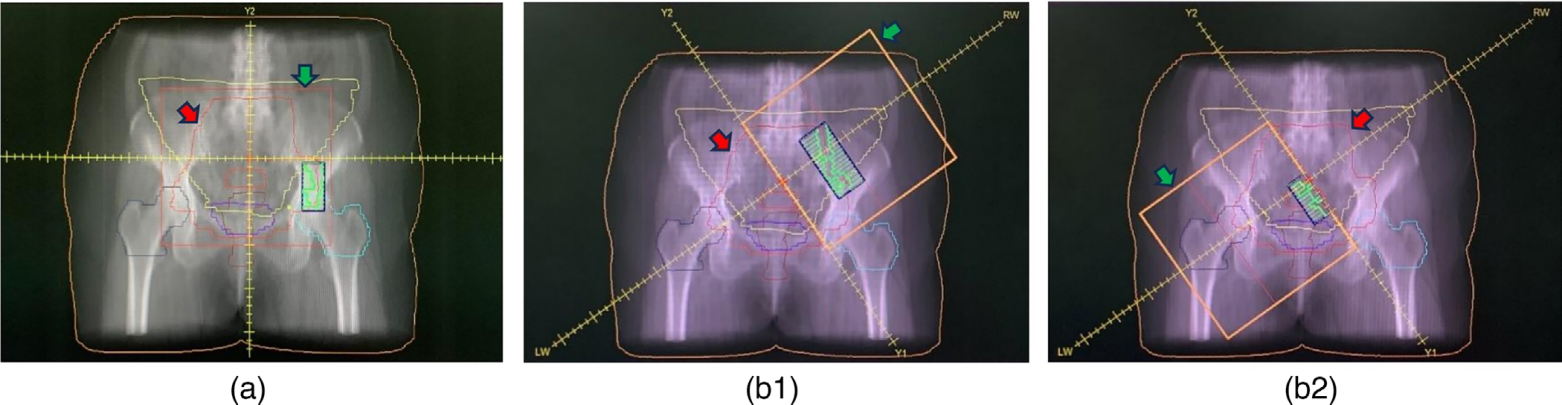
The Beam's Eye View (BEV) G180° of the AF‐VMAT (A) consists of two full coplanar arcs, one rotating clockwise and the other counterclockwise, without collimator rotation and with the auto field setup. The MF‐VMAT consists of two full coplanar arcs with the 35° collimator rotation and the fixed field setup, one rotating clockwise with the left x‐jaw shielding (b1) and the other counterclockwise with the right x‐jaw shielding (b2). The PTV contour (red arrow), the field size opening (green arrow), and OARs including the bowel bag (yellow line), rectum (brown line), bladder (purple line), left femoral head (dark blue line), right femoral head (light blue line), and whole‐body contour (dark yellow line). AF‐VMAT, auto field volumetric modulated arc therapy; MF‐VMAT, modified fixed field volumetric modulated arc therapy.

In the AF‐VMAT technique, the treatment planning system (TPS) automatically opened the jaws according to the target, with a collimator angle of 0°, and auto fields were used in both arcs as in Figure 1a. Conversely, in the MF‐VMAT technique, the field size was manually opened by fixing the jaws, with a collimator angle of 35°, and fixed fields were used in both arcs as referenced from the previous study.^34^ Each arc contains one half‐beam field, with either the left or right side shielded by the x‐jaw as also referenced from former studies on half‐beam VMAT in pelvic cancer.^18^, ^19^, ^20^, ^21^ For the fixed field setup, the x‐jaw opening was set at 20 × 20 cm^2^ (fully opened) because our preliminary study has shown that a reduced x‐jaw opening will result in worse dosimetric results for PTV compared to a fully opened x‐jaw. The y‐jaw opening was maintained at 1 cm from the PTV margin as in Figure 1b1,b2, to ensure that the field size opening covers the entire PTV volume and to compensate for the penumbra of centered fields (6 and 10 MV), which is less than 6 mm at d_max_ for field sizes ranging from 15 × 15 cm^2^ to 35 × 35 cm^2^ in the Elekta Synergy LINAC. The prescription dose was 46 Gy delivered in 2 Gy per fraction to the PTV for a total of 56 treatment plans and all plans in both treatment techniques used the 100% dose normalization. The target contouring was performed according to the EMBRACE II and Radiation Therapy Oncology Group (RTOG) guidelines, ensuring that the PTV included the tumor and regional lymph nodes. After optimization and dose calculation, the dose was normalized such that 100% of the prescribed dose covered 100% of the PTV volume.

We utilized a 6 MV photon beam from the Elekta Synergy linear accelerator (Elekta, Stockholm, Sweden), which is equipped with an agility 160 MLC featuring a 5 mm leaf width projected at the isocenter for treatment delivery. For treatment planning, we employed the Monaco version 6.1.3.0 TPS (Elekta, Stockholm, Sweden), with the Monte Carlo algorithm and 3 mm grid size for dose calculation.

### 2.3 Planning objectives and optimization

The overlap priority of OARs for all planning optimizations was as follows: bowel bag, rectum, bladder, right femoral head, and left femoral head, respectively. The same cost functions, dose constraints, and overlap priorities were applied to both treatment planning techniques in each patient. Dose constraints for the target were evaluated based on the guidelines provided by the International Commission on Radiation Units and Measurements (ICRU 83). For all treatment plans, the PTV was optimized to achieve the following parameters: V_107%_ ≤ 2%, V_100%_ ≥ 50%, and V_95%_ ≥ 98%. Where the V_107%_, V_100%_, and V_95%_ were referred to the volumes of PTV that received doses corresponding to 107%, 100%, and 95% of the prescription dose, respectively. Additionally, PTV parameters including V_107%_, V_100%_, V_95%_, conformity index (CI), and homogeneity index (HI) were controlled for maintaining statistically insignificant between both treatment planning techniques for OARs dose statistic comparison. The CI was calculated as the ratio of the volume of PTV that received 95% of the prescription dose squared to the total volume of PTV, multiplied by the volume of the external contour that received 95% of the prescription dose. A value of CI close to one is considered indicative of a conformal plan.

$$\mathrm{CI}=\frac{T{V_{\mathrm{PIV}}}^{2}}{\mathrm{TV}\times \mathrm{PIV}}$$

where TV_PIV_ is the volume of PTV in cm^3^ which received 95% of the prescription dose, TV is the volume of PTV in cm^3^, and PIV is the volume of the whole‐body in cm^3^ which received 95% of the prescription dose.

The HI was calculated as the ratio of the difference between D_2%_ and D_98%_ to D_50%_, which refers to the dose received by 2%, 98%, and 50% of the PTV, respectively. A value of HI close to zero is considered indicative of a homogeneous plan.

$$\mathrm{HI}=\frac{\left(D_{2\%}-D_{98\%}\right)}{D_{50\%}}$$

The OARs were optimized to achieve the parameters based on the Radiation Therapy Oncology Group (RTOG 0418 and 1203) guidelines, including bowel bag: up to 30% receives 40 Gy, rectum: up to 80% receives 40 Gy, bladder: up to 35% receives 45 Gy, and femoral head: no more than 15% receives 30 Gy.

### 2.4 Planning techniques comparison

#### 2.4.1 Dosimetric and treatment plan quality comparison

Both treatment planning techniques were evaluated based on dose‐volume statistics and plan quality indices. Regarding the PTV parameters, V_107%_, V_100%_, V_95%_, CI, and HI were maintained as statistically insignificant and met the criteria outlined in ICRU83. For the OARs parameters, including bowel bag V_40Gy_, rectum V_40Gy_, bladder V_45Gy_, right femoral head V_30Gy_, and left femoral head V_30Gy_ and whole‐body V_20Gy_ were compared between both techniques. The whole‐body encompassed the entire CT scan volume, which was used to evaluate the low dose spread to normal tissues between both techniques.

#### 2.4.2 Treatment plan efficiency

To assess plan efficiency, the following parameters were evaluated: number of MUs, number of control points, beam‐on time, maximum leaf travel, average maximum leaf travel, and maximum leaf travel per gantry rotation. These parameters were calculated in Monaco TPS and displayed in the optimization console.

#### 2.4.3 Treatment plan accuracy

Furthermore, patient‐specific quality assurance (PSQA) was performed by the new Model 1220 ArcCHECK (Sun Nuclear Corporation, Melbourne, Florida, USA) and SNC patient software version 8.5.1.9 to evaluate the delivery accuracy of the plans. Both AF‐VMAT and MF‐VMAT plans were delivered using the Elekta Synergy linear accelerator on the same day. The gamma passing rates (GPR) with a 10% dose threshold were determined for both AD and relative dose (RD) using gamma criteria of 3%/3 and 3%/2 mm, respectively.

### 2.5 Statistical analysis

All results data were tested for normality. If the data followed a normal distribution, the two‐tailed paired samples *t*‐test was conducted to compare the results between AF‐VMAT and MF‐VMAT plans. If the data did not follow a normal distribution, the Wilcoxon signed‐rank test was used to compare the results between both treatment planning techniques. Data analysis was carried out using IBM SPSS Statistics version 26.0.0.1, with *p* < 0.05 considered statistically significant.

## 3 RESULTS

### 3.1 Dosimetric and treatment plan quality comparison

#### 3.1.1 PTV

**FIGURE 2:**
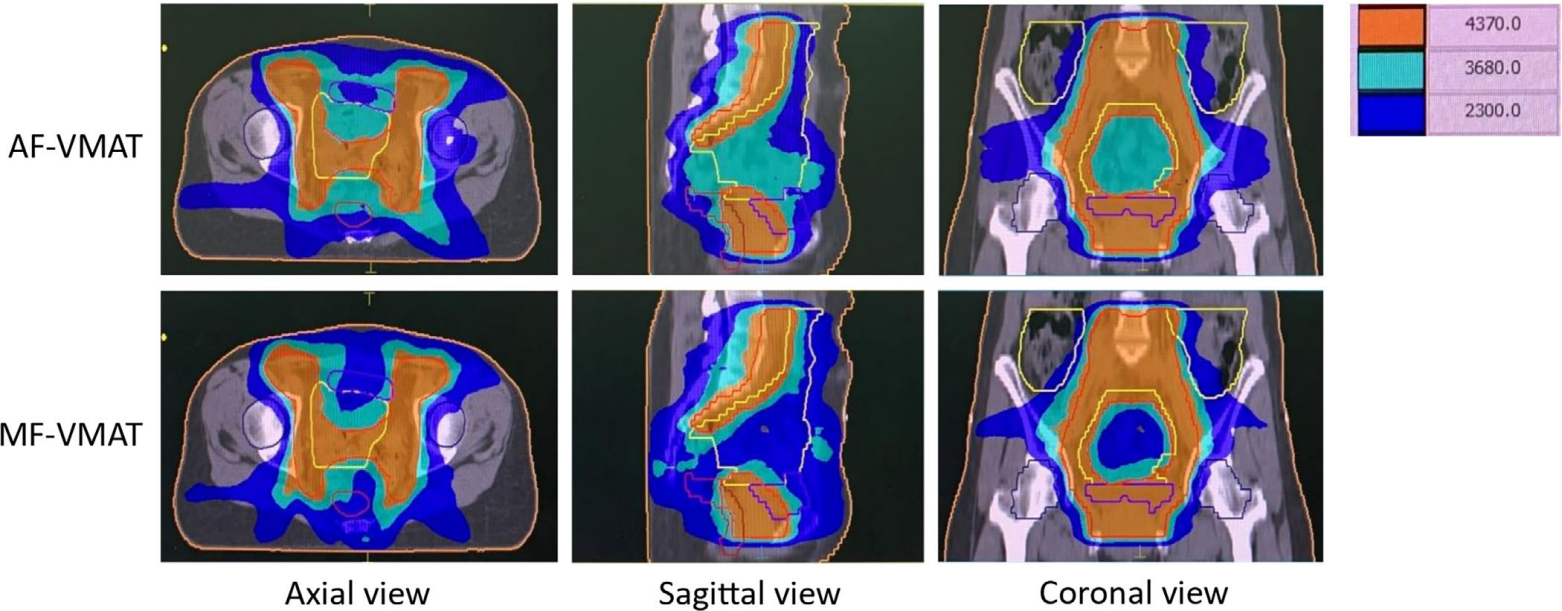
Dose distribution comparison between AF‐VMAT and MF‐VMAT. AF‐VMAT, auto field volumetric modulated arc therapy; MF‐VMAT, modified fixed field volumetric modulated arc therapy.

Figure 2 demonstrates the dose distribution comparison between AF‐VMAT and MF‐VMAT. The dosimetric parameters of the PTV and plan quality indices including V_107%_, V_100%_, V_95%_, CI, and HI were maintained as statistically insignificant between both treatment planning techniques for each patient. Table 2 demonstrates the PTV dose, CI, and HI for the two techniques were comparable with no significant difference by *p*‐value 0.263, 0.062, and 0.157 for V_100%_, CI, and HI, respectively.

**TABLE 2:** Dosimetric parameters and plan quality indices of the PTV.

	AF‐VMAT	MF‐VMAT	
Parameters	Mean ± SD	Mean ± SD	*p*‐value
V_107%_ (%)	0.06 ± 0.05	0.06 ± 0.06	0.586
V_100%_ (%)	75.69 ± 5.29	75.40 ± 4.98	0.263
V_95%_ (%)	98.73 ± 0.50	98.69 ± 0.48	0.428
CI	0.83 ± 0.04	0.83 ± 0.04	0.062
HI	0.09 ± 0.01	0.09 ± 0.01	0.157

Abbreviations: AF‐VMAT, auto field volumetric modulated arc therapy; MF‐VMAT, modified fixed field volumetric modulated arc therapy; SD, standard deviation.

#### 3.1.2 OARs and whole‐body

Table 3 demonstrates the dosimetric parameters of the OARs including bowel bag V_40Gy_, rectum V_40Gy_, bladder V_45Gy_, right femoral head V_30Gy_, and left femoral head V_30Gy_ and whole‐body V_20Gy_ between both treatment planning techniques. The results showed that the bowel bag (*p*‐value = 0.001), rectum (*p*‐value = 0.002), left femoral head (*p*‐value = 0.001), and whole‐body (*p*‐value = 0.000) received a statistically significant dose reduction in the MF‐VMAT plan compared to the AF‐VMAT plan. The average volume that received the specified dose was 21.15 ± 10.25%, 54.63 ± 14.51%, 1.00 ± 1.56%, and 29.01 ± 7.86%, respectively, indicating that MF‐VMAT could spare more OARs than AF‐VMAT.

**TABLE 3:** Dosimetric parameters of OARs and whole‐body.

Parameters	AF‐VMAT Mean ± SD	MF‐VMAT Mean ± SD	*p*‐value
Bowel bag: V_40Gy_ (%)	22.87 ± 11.24	21.15 ± 10.25	0.001^*^
Rectum: V_40Gy_ (%)	56.13 ± 14.58	54.63 ± 14.51	0.002^*^
Bladder: V_45Gy_ (%)	37.64 ± 15.73	38.17 ± 14.98	0.273
Rt. HOF: V_30Gy_ (%)	1.09 ± 1.08	1.12 ± 1.42	0.230
Lt. HOF: V_30Gy_ (%)	1.42 ± 1.50	1.00 ± 1.56	0.001^*^
Whole‐body: V_20Gy_ (%)	30.23 ± 8.21	29.01 ± 7.86	0.000^*^

Abbreviations: AF‐VMAT, auto field volumetric modulated arc therapy; Lt. HOF, left head of the femur; MF‐VMAT, modified fixed field volumetric modulated arc therapy; Rt. HOF, right head of the femur; SD, standard deviation.

* Statistical significance.

### 3.2 Treatment plan efficiency

**FIGURE 3:**
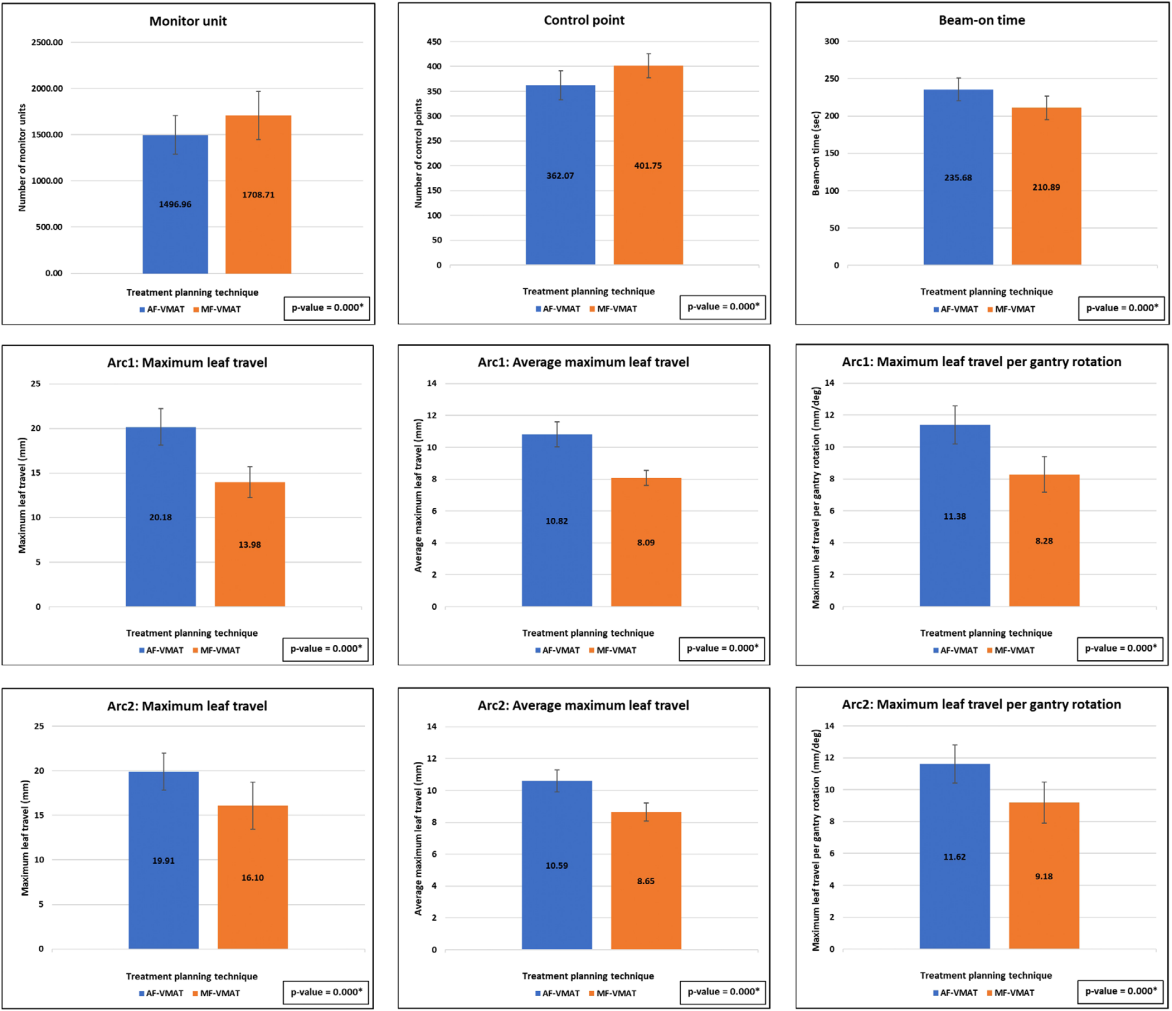
Comparison of plan efficiency parameters between AF‐VMAT and MF‐VMAT. *Statistical significance. AF‐VMAT, auto field volumetric modulated arc therapy; MF‐VMAT, modified fixed field volumetric modulated arc therapy.

Figure 3 demonstrates the parameters used for plan efficiency evaluation, including the number of MUs, the number of control points, beam‐on time, maximum leaf travel, average maximum leaf travel, and maximum leaf travel per gantry rotation. The results showed a statistically significant increase with *p*‐values of 0.000 in both the number of MUs and the number of control points when using the MF‐VMAT, while beam‐on time, maximum leaf travel, average maximum leaf travel, and maximum leaf travel per gantry rotation were statistically significant decreased with *p*‐values of 0.000 in all above parameters compared to the AF‐VMAT.

### 3.3 Treatment plan accuracy

Table 4 demonstrates the parameters used for plan accuracy evaluation. All VMAT plans passed the 3%/3 mm gamma criteria based on TG 148, which states that the GPR should be ≥ 90%. Only one case in AF‐VMAT did not meet the action level of a GPR ≥ 90% for the RD of 3%/2 mm gamma criteria based on TG 218.^35^ Under the 3%/3 mm gamma criteria, the average GPR in the MF‐VMAT plan was 98.45 ± 0.59% and 98.47 ± 0.67% for absolute and RD, respectively. This was higher than that of the AF‐VMAT plan, with averages of 97.76 ± 1.13% and 97.81 ± 1.17% for absolute and RD, respectively, showing statistical significance (AD: *p*‐value = 0.001, RD: *p*‐value = 0.000). For the 3%/2 mm gamma criteria, the average GPR in the MF‐VMAT plan was 96.88 ± 1.18% and 96.95 ± 1.19% for absolute and RD, respectively. This was higher than that of the AF‐VMAT plan, with averages of 96.01 ± 1.79% and 95.85 ± 1.92% for absolute and RD, respectively, also showing statistical significance (AD: *p*‐value = 0.004, RD: *p*‐value = 0.000).

**TABLE 4:** The average gamma passing rate with 3%/3 and 3%/2 mm gamma criteria for both absolute and relative dose.

Gamma passing rate	Dose parameter	AF‐VMAT Mean ± SD	MF‐VMAT Mean ± SD	*p*‐value
3%/3 mm gamma criteria	AD	97.76 ± 1.13	98.45 ± 0.59	0.001^*^
	RD	97.81 ± 1.17	98.47 ± 0.67	0.000^*^
3%/2 mm gamma criteria	AD	96.01 ± 1.79	96.88 ± 1.18	0.004^*^
	RD	95.85 ± 1.92	96.95 ± 1.19	0.000^*^

Abbreviations: AD, absolute dose; AF‐VMAT, auto field volumetric modulated arc therapy; MF‐VMAT, modified fixed field volumetric modulated arc therapy; RD, relative dose; SD, standard deviation.

* Statistical significance.

## 4 DISCUSSION

According to the beam geometry of MF‐VMAT, which includes the use of fixed jaws to shield one hemi‐side, with a collimator angle of 35° and two full coplanar arcs, MF‐VMAT can generate a double concave dose distribution relative to the geometry of PTV in cervical cancer.^18^ Furthermore, it demonstrates the advantages of reducing OAR doses and improving the low‐dose spread to normal tissues. With this treatment planning technique, the MLCs do not need to frequently move across the midline,^18^ meaning that the leaf speed is reduced, therefore better optimizing the dose to PTV and minimizing the dose to OARs. Half‐beam fields are crucial for generating the double concave dose distribution as they reduce beam divergence and aid in beam focusing.^18^ Previous studies on half‐beam VMAT in pelvic cancer have shown the advantages of reducing the dose to OARs and facilitating a better PTV dose.^18^, ^19^, ^20^, ^21^

Due to the 5‐year OS rate for both early‐stage and locally advanced‐stage cervical cancer patients being more than 60%,^10^, ^11^, ^12^, ^13^ the radiation‐induced toxicity for many OARs in gynecological cancer following pelvic radiotherapy is a significant concern. GI radiation‐induced toxicity is a major complication ranging from mild to very severe in non‐cancerous tissues resulting from radiotherapy to a pelvic tumor, also known as pelvic radiation disease.^36^ Despite recent improvements in radiation techniques, acute and late radiation‐induced GI toxicity is still frequently observed.^36^ The 5‐year cumulative incidence was 9.2% for late GI and GU toxicities of grade ≥ 3 and 51.8% for acute leukopenia of grade ≥ 3.^37^ Pelvic insufficiency fractures (PIFs) induced by radiation therapy for gynecological cancers are frequently observed in postmenopausal patients within 1 year after external beam radiation therapy (EBRT).^38^ Post‐IMRT PIFs were detected in 18.4% of patients with LACC,^39^ while the 5‐year risk of PIFs for postoperative pelvic radiation therapy in cervical and endometrial cancer is 5.1%.^40^

**FIGURE 4:**
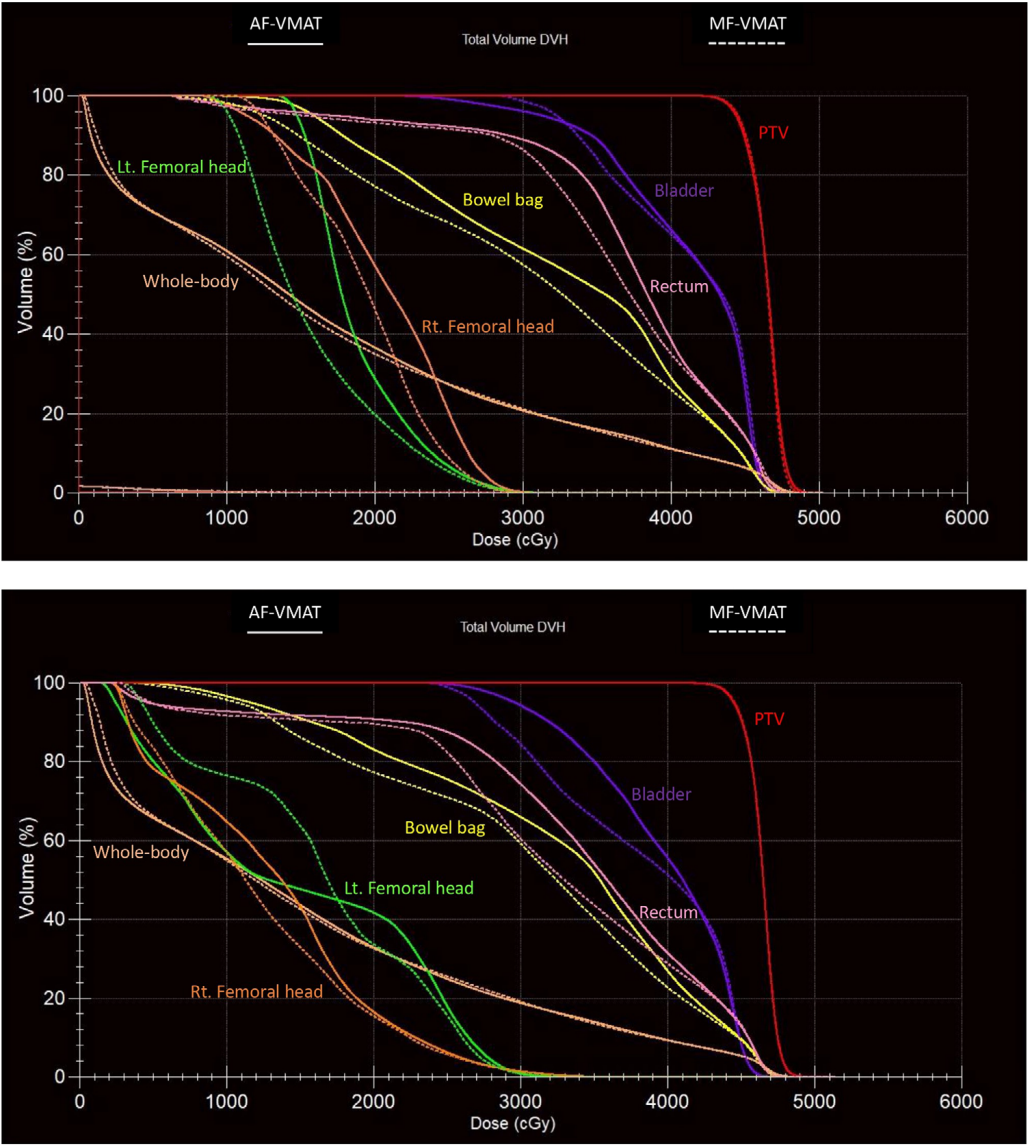
Comparison of the dose volume histogram for two patients between AF‐VMAT and MF‐VMAT. AF‐VMAT, auto field volumetric modulated arc therapy; MF‐VMAT, modified fixed field volumetric modulated arc therapy.

In this study, we maintained the PTV parameters: V_107%_, V_100%_, V_95%_, CI, and HI with statistically insignificant differences and compared AF‐VMAT with MF‐VMAT in three parts: dosimetric and plan quality comparison, plan efficiency, and plan accuracy. As shown in Figure 2, the dose distribution between the two techniques reveals that 80% of the prescription dose (36.8 Gy) in the blue area is more conformal to the PTV (red line) when using MF‐VMAT. The MF‐VMAT provides sharper dose fall‐off with a separation of 80% of the prescription dose between the left and right sides of the PTV. The axial view also displays a double‐concave dose distribution in MF‐VMAT, resulting in sculpted dose curvatures along the posterior bladder wall and anterior rectal wall, relative to the former studies.^18^, ^19^ For the dosimetric parameters of OARs and whole‐body, MF‐VMAT achieves statistically significant dose reduction in bowel bag V_40Gy_, rectum V_40Gy_, Lt. HOF V_30Gy_, and whole‐body V_20Gy_, with *p*‐values of 0.001, 0.002, 0.001, and 0.000, respectively. Only the bladder does not meet the dose constraint based on RTOG 0418 guidelines, which state that no more than 35% should receive a dose of 45 Gy in both techniques (AF‐VMAT: 37.64 ± 15.73%, MF‐VMAT: 38.17 ± 14.98%). This discrepancy may be attributed to the fact that almost all patients followed an empty bladder protocol, resulting in the bladder being closer to the PTV and receiving a higher dose volume from the PTV. For the femoral head, only the left femoral head receives significant dose reduction when using MF‐VMAT. This difference may be attributed to the beam geometry design of MF‐VMAT, which rotates the collimator to 35°, causing the field size to fully cover the left femoral head compared to the right femoral head. However, the half‐beam design of MF‐VMAT leads to a reduced dose to the left femoral head compared to AF‐VMAT. The parameter whole‐body V_20Gy_ is used to compare the low dose spread between both techniques.^18^ The results from Table 3 show a statistically significant dose reduction when using MF‐VMAT, relative to the former studies.^18^, ^19^ The dose‐volume histogram for two patients shows better sparing of OARs when using MF‐VMAT, as shown in Figure 4. Therefore, MF‐VMAT achieves a statistically significant reduction in OARs dose and decreases the spread of low doses for cervical cancer patients. This advantage stems from dividing the field size into two half‐beam fields, with each field focusing on one hemi‐side of the optimization area. This increases the efficiency in sculpting the dose to conform to the PTV while minimizing the dose to OARs with jaw shielding.^18^

Regarding the average MUs reflecting the modulation of the plans, plans with higher MUs indicate increased plan modulation capability.^21^, ^41^ The MF‐VMAT has significantly higher average MUs and control points than AF‐VMAT, with *p*‐values of 0.000. This increase in average MUs is relative to previous studies.^18^, ^19^, ^20^, ^21^ Treatment plans with increased MUs typically result in longer treatment times.^18^, ^20^, ^21^, ^42^ However, this study demonstrates that MF‐VMAT achieves lower beam‐on times compared to AF‐VMAT, with a statistical significance at a *p*‐value of 0.000. The lower beam‐on time while higher MUs in MF‐VMAT may result from the half‐beam design. The MF‐VMAT was separately optimized with each side of the PTV volume by half field irradiated. It is brought to a shorter distance of leaf travel and shorter overall beam‐on time as in Figure 3. Two coplanar arcs of MF‐VMAT are equivalent to a single optimization of the full PTV, while AF‐VMAT is equivalent to two optimizations of the full PTV. Although several studies have reported the advantages of half‐beam VMAT in reducing the dose to OARs and facilitating a better PTV dose,^18^, ^19^, ^20^, ^21^ one study reported that half‐beam VMAT used two times higher MUs than full‐beam VMAT, but with identical beam‐on time and more precise MLCs leaf motion.^19^ Another study reported that half‐beam VMAT resulted in an increase in MUs and treatment time compared to open and limited beam VMAT, but they used four arcs in half‐beam compared to two arcs in open and limited planning techniques.^20^, ^21^ The other study reported that half‐beam VMAT resulted in an increase in MUs and treatment time compared to full‐beam VMAT with the same number of arcs.^18^

VMAT delivers modulated photon beam intensity by simultaneously modulating MLCs position, gantry speeds, and dose rates at each control point.^42^, ^43^ MLCs are utilized to shield anatomical structures from photon radiation and to modulate the incident photon fluence field.^44^ Highly modulated dose distributions commonly require MLCs to move at high speeds during gantry rotation.^45^, ^46^, ^47^ However, fast leaf motion during gantry rotation may be affected by interleaf friction or MLC motor problems, resulting in leaf position errors.^45^, ^48^, ^49^ In VMAT techniques, both the mean and maximum leaf speeds were significantly associated with the leaf root mean square (RMS) error. To enhance VMAT treatment and MLC performance by reducing the leaf RMS error, it is recommended to lower the mean and/or maximum leaf speeds.^22^ In this study, we recorded the parameters for both arcs, including maximum leaf travel, average maximum leaf travel, and maximum leaf travel per gantry rotation, which are relative to leaf speed. The results from Figure 3 show that MF‐VMAT achieves lower average values for all these parameters in both arcs compared to AF‐VMAT, with statistical significance at *p*‐values of 0.000. The most significant average difference among the parameters in both techniques is the maximum leaf travel, which is 6.20 and 3.81 mm in arc 1 and arc 2, respectively. The half‐beam design of MF‐VMAT directly relates to a decrease in the maximum distance of the leaf.

The modulation of MLCs movement is a critical factor influencing the plan delivery accuracy of VMAT.^23^, ^24^, ^25^, ^26^ One study has shown that as MLCs speed and acceleration increase, both global and local GPRs decrease, MLCs positional errors increase, and the magnitude of changes in dose‐volumetric parameters increases, indicating a decrease in plan delivery accuracy.^27^ As shown in a previous study, restricting the maximum MLCs speed can improve VMAT delivery accuracy.^22^ In this study, MF‐VMAT achieves a higher average GPR in both AD and RD using the 3%/3 mm gamma criteria compared to AF‐VMAT, with statistical significance at *p*‐values of 0.001 and 0.000, respectively. This trend is also observed for the 3%/2 mm gamma criteria, MF‐VMAT exhibiting a higher average GPR than AF‐VMAT, with statistical significance at *p*‐values of 0.004 and 0.000 in AD and RD, respectively, as reported in Table 4. Therefore, decreasing the maximum leaf travel of MF‐VMAT in this study leads to an increase in the GPR relative to the previous studies.^22^, ^27^

## 5 CONCLUSION

MF‐VMAT is an effective treatment planning technique for cervical cancer. The beam geometries designed for MF‐VMAT, provide significant reductions in OAR doses and decrease the spread of low doses to normal tissues. Half‐beam fields in MF‐VMAT are essential for generating the double concave dose distribution, which reduces the maximum MLCs travel, relative to MLCs speed, indicating an increase in plan delivery accuracy through an increase in the average GPR for both 3%/3 and 3%/2 mm gamma criteria. Although MF‐VMAT has higher MUs and control points, it demonstrates a significant decrease in beam‐on time compared to AF‐VMAT. Therefore, MF‐VMAT offers advantages in dosimetric comparison, plan quality, plan efficiency, and plan delivery accuracy for cervical cancer VMAT treatment planning.

## CONFLICT OF INTEREST STATEMENT

The authors declare no conflicts of interest.
